# Metal–organic frameworks-based biosensor for microRNA detection in prostate cancer cell lines†

**DOI:** 10.1039/d2ra04959g

**Published:** 2022-12-07

**Authors:** Milad Ahmadi Najafabadi, Fatemeh Yousefi, Mohammad J. Rasaee, Masoud Soleimani, Mahmood Kazemzad

**Affiliations:** Department of Medical Biotechnology, Faculty of Medical Sciences, Tarbiat Modares University Tehran Iran rasaee_m@Modares.ac.ir; Department of Department of Genetics, Faculty of Biological Sciences, Tarbiat Modares University Tehran Iran; Department of Hematology, Faculty of Medical Sciences, Tarbiat Modares University Tehran Iran Soleimani.masoud@gmail.com; Material & Energy Center (MERC) Tehran Iran

## Abstract

In this research, a novel dye-labeled probe (FAM-Probe) based on a nano metal–organic framework (NMOF) functionalized with folate (NMOF-FA) was prepared and applied as a fluorescent sensing platform for the recognition of intracellular microRNA (miRNA-21) in DU145, PC3, and LNCaP cancer cells. The NMOF-FA can be easily assembled with a dye-labeled miR-21 probe (FAM-Probe21), causing an efficient fluorescence quenching of fluorescence of FAM fluorophore. The probe can be specifically catch up by cancerous cells through targeting their folate receptor by folic acid on the FAM-Probe21-NMOF-FA complex. Upon the interaction of the FAM-Probe21-NMOF-FA with complementary miRNA (miR-21), the fluorescence intensity can be recovered, providing a specific system to detect miRNAs in prostate cancer cells. We used the proposed probe for cell-specific intracellular miRNA-21 sensing, following the alteration expression level of miRNA-21 inside living cells. Thus, the FAM-Probe21-NMOF-FA complex can be used as a new miRNA sensing method in biomedicine studies.

## Abstract 2

A novel FAM-Probe based on a nano metal–organic framework was functionalized with folate and applied to detect the intracellular miR-21 in prostate cancer cells. Upon the interaction of the probe with complementary miRNA, the fluorescence intensity can be recovered.

## Graphical abstract

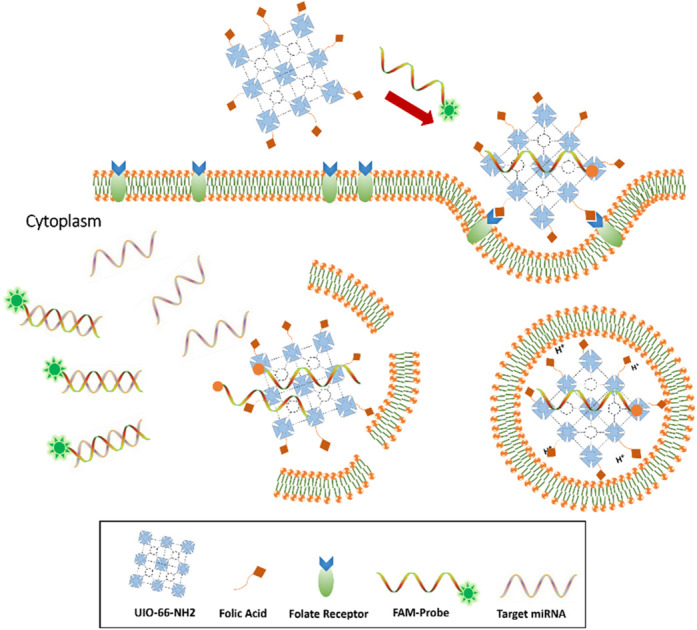

## 1. Introduction

Prostate cancer (PCa) remains the most common type of malignancy in men and the third leading cause of cancer-related death worldwide.^1^ The problematic early detection of PCs is mainly caused by the high mortality rate in patients with prostate cancer. So, it is an immediate need to develop rapid and early diagnostic strategies for PCa to enhance the survival rate of patients.

microRNAs (miRNAs) are a numerous class of small non-protein-coding RNAs that are about 22 nucleotides in length and modulate cellular signaling pathways through mediating gene expression.^2,3^ miRNAs play post-transcriptional regulation through mRNA degradation or translational inhibition. Therefore, they have a pivotal role in a vast variety of biological processes, including cellular differentiation, early development, proliferation, and apoptosis.^4–6^ The misregulation and aberrant expression of miRNAs are associated with a large number diseases, including human cancers.^7–9^ So, developing a sensing strategy for quantitatively measure of the expression level of multiple miRNAs can be useful for prognosis and diagnosis in clinical procedures. Today, many techniques have been used for miRNA analysis, such as northern blotting,^10,11^ real-time quantitative PCR,^12,13^ and microarrays,^14,15^ which all require a miRNA extraction step for sample preparation and/or target amplification. Whereas, quantitative monitoring of intracellular miRNAs remains a significant challenge, and developing an *in situ* monitoring technique for detecting miRNAs in living cells seems necessary.

Recently, fluorescence sensors have been a broad concern in biomolecule detection cause of their great sensitivity, PCR-free sensing platforms, ease of operation, and online cellular imaging.^16,17^ Fluorescence sensors are usually based on fluorescence quencher of dye-labeled DNA probes and function through a fluorescence resonance energy transfer (FRET) system. Nanomaterials have been widely used in fluorescence sensors due to their great fluorescence quenching capability.^18,19^ For example, carbon nanomaterials, including graphene oxide (GO)^20^ and carbon nanotubes (CNTs)^21^ are introduced as the most common fluorescence quenchers.

**Fig. 1:**
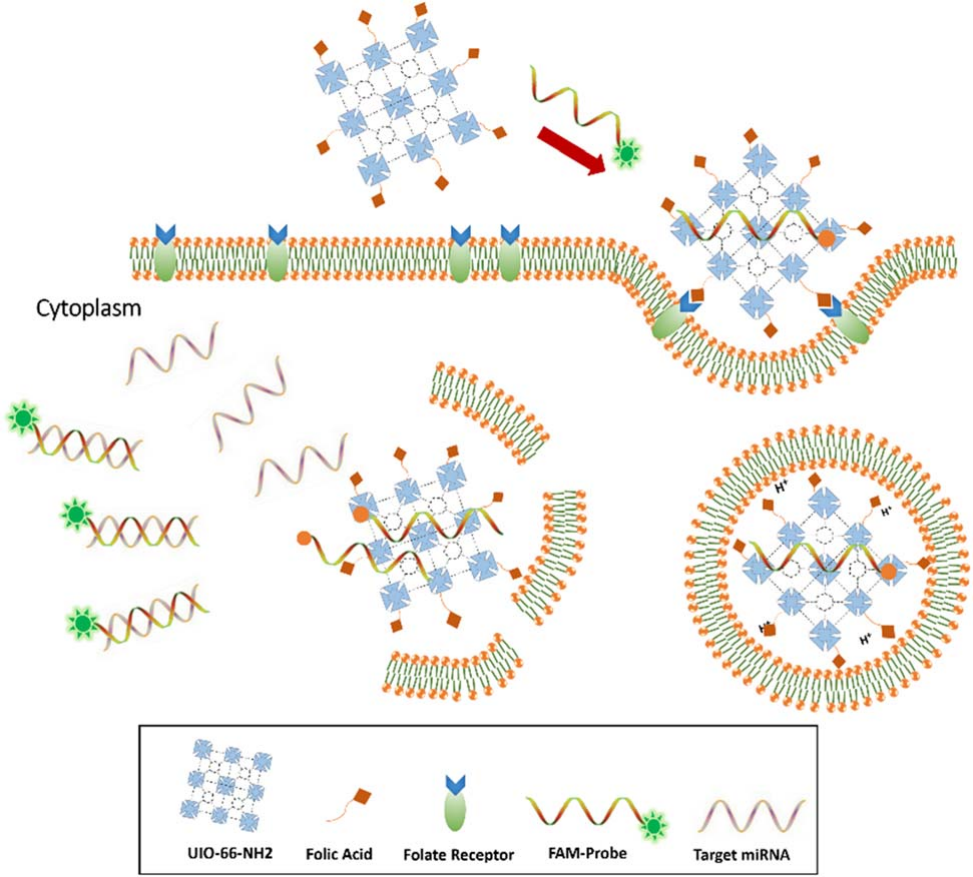
Sensing mechanism of the one-step *in situ* detection of miRNA-21 expression in single cancer cells based on UIO-66-NH_2_ probe.

Metal–organic frameworks (MOFs) are mesoporous hybrid substances composed of metal ions and some clusters connected by organic linker groups in suitable solvents, which have been a widespread concern in recent years. Compared to conventional nanostructures, NMOFs have various unique properties, such as flexible porosity, tunable structures, and large surface area.^22,23^ These unique properties of MOFs enable themselves to apply in gas storage,^24–26^ small molecule separation,^27,28^ imaging,^29,30^ heterogeneous catalysis,^31^ and drug delivery.^32,33^ Because of porous property and intrinsic fluorescence quenching, MOFs can construct fluorescence sensor to capture and detect small molecules, such as various miRNAs,^34^ pathogen DNAs, and antibodies in serum samples.^35,36^ In this study, we provide an innovative miRNA sensing method based on NMOF-FA composed of a DNA probe for detecting of miR-21 as a biomarker in prostate cancer cell lines (DU145, PC3, and LNCaP). The FAM-Probe21-NMOF-FA complex was prepared by assembling FAM-Probe21 and NMOF-FA through strong π–π interactions.^37^ In detail, when the DNA probes are tightly attached to the MOF carrier, the fluorescence of dye-labeled is extremely quenched due to photo-induced electron transfer (PET) processes,^36^ and the presence of FA on NMOF led to cell-target-specific delivery.^38^ In the presence of miR-21 (as a target), the FAM-labeled DNA probes are released from the MOF and hybridize with the target miRNAs in the cells, causing the fluorescence emission of DNA probes (Fig. 1).

## 2. Material and methods

### 2.1. Synthesis of NMOF (UiO-66-NH_2_)

Briefly, 0.08 g zirconium(iv) chloride (ZrCl_4_), 0.073 g 2-amino terephthalic acid (NH_2_-H_2_BDC), and 0.316 g benzoic acid was mixed into 20 mL dimethylformamide (DMF). The mixture was then sonicated for 20 min and reacted at 120 °C for 24 h. The obtained solids were separated by centrifugation (9000 rpm, 10 min) after cooling down to room temperature, then washed with DMF (2 × 20 mL) and methanol (3 × 20 mL), and dried in a vacuum oven under 60 °C.

### 2.2. Preparation of the FAM-Probe21-NMOF-FA complex

Briefly, 0.2 g FA, and 0.1 g UIO-66-NH_2_ were mixed into an aqueous solution (10 mL). Then, 0.1 g *N*-(3-dimethyl aminopropyl)-*N*-ethyl carbodiimide hydrochloride (EDC) was added to the mix, which was subsequently stirred in the dark at room temperature for 16 h to allow the FA to conjugate onto the UIO-66-NH_2_. The obtained UIO-66-NH_2_-FA nanostructure was isolated from the solution by centrifugation (8000 rpm, 10 min), followed by washing with water and kept in water for biosensing study and then dried at room temperature. To prepare FAM-Probe21-NMOF-FA complex and FAM-ScProbe-NMOF-FA complex, FAM-Probe21 or FAM-ScProbe was mixed into NMOF-FA (in PBS, pH = 7.2) for 20 min at room temperature and subsequently used for studying.

### 2.3. Apparatus and measurements

The morphology and size of NMOF (UiO-66-NH_2_), NMOF-FA, and FAM-Probe21-NMOF-FA complex were characterized by a field emission scanning electron microscope (FE-SEM ZEISS Sigma 300). The EDX spectrum was obtained on a ZEISS Sigma 300. Dynamic light scattering (DLS) was performed to measure size distribution of nanoparticles by using Malvern Zetasizer Nano-ZS ZEN 3600. The BET surface area was characterized by N_2_ adsorption–desorption isotherms at 77 K using a Micromeritics TriStar II Version 3020 3.02. An X-ray diffraction patterns were recorded on an X-ray diffractometer (X'Pert MPD, Cu radiation, Netherlands). A fluorometer (SynergyMx, Biotek, U.K.) was used for recording fluorescence emission. The cell images were obtained by a TCS SP5 laser scanning confocal microscope (Leica, Germany). The fluorescence intensity of each sample was measured by a FACSCalibur flow-cytometer (BD Bioscience; USA). RT-qPCR was performed by using ABI 7500 fast real-time PCR system (Applied Biosystems, Foster City, CA, USA). The cell viability was measured with an ELISA Microplate Reader (Biotek).

### 2.4. Cell culture

Three prostate cancer cell lines in different stages (DU145, PC3, and LNCaP) and one lung cancer epithelial cell line (A549) were used in this study. Cell lines were purchased from the Pasteur Institute (Tehran, Iran). The cells were cultured in Roswell Park Memorial Institute Medium (RPMI) (Gibco; USA) and supplemented with 10% (v/v) fetal bovine serum (Gibco; USA) with 1% antibiotics (100 U mL^−1^ of penicillin and 100 μg mL^−1^ of streptomycin) (Gibco; USA) at 37 °C in a humidified atmosphere of 5% CO_2_.

### 2.5. Transfection

To alter the expression of miR-21, cells were cultivated in 48-well plates at a density of 3 × 10^5^ cells per well, 24 h before treatment. Then, cells were treated with 250 μL serum-free RPMI and PEI (polyethylenimine) containing 50 nM antisense-21, or 5 nM miRNA-21 mimic for 24 h at 37 °C. Afterward, these cells were treated with 10 μg per mL FAM-Probe21-NMOF-FA at 37 °C for 4 h. After washing with PBS, these cells were used for confocal imaging analysis to miRNA detection.

### 2.6. miRNA detection in solution

FAM-Probe21-NMOF complex (3 μg per mL NMOF and 5 μM FAM-Probe21) was blended into miR-21 target in different concentrations (0–1000 nM) to check complex sensitivity, and also in another set of experiments, the solution was blended with different target miRNA samples (miR-21, miR-155, miR-16) to evaluate specificity and cross-reactivity of the complex. Then, to examine the fluorescence emission, a fluorimeter (SynergyMx, Biotek, U.K.) was carried out. The quenching efficiency QE was calculated upon the presence (FM) and absence (*F*_0_) of NMOF *via* QE = (1 − FM/*F*_0_) × 100%. Recovery efficiency (RE) of fluorescence was calculated as RE = (FT/FM − 1) × 100%, wherein FM and FT are the fluorescence intensities at 520 nm in the absence and presence of the targets after introducing an NMOF, respectively. Each experiment was performed in triplicate.

### 2.7. Cytotoxicity assay of FAM-Probe21-NMOF-FA complex (MTT assay)

MTT assay was applied to investigate of FAM-Probe21-NMOF-FA complex cytotoxicity in DU145, PC3, and LNCaP cells. Briefly, 1.5 × 10^4^ DU145, PC3 and, LNCaP cells were seeded in 96-well plates in tetraplicate and incubated for 24 h at 37 °C with 5% CO_2_. These cells were incubated with different concentrations of FAM-Probe21-NMOF-FA complex (0–100 μg mL^−1^) for 24 h. And added with 50 μL MTT (1 mg mL^−1^) then incubated for two hours. The culture media was discarded, and 150 μL dimethyl sulfoxide (DMSO) was added to dissolve the formed formazan dye. Finally, the function of cell viability was measured with an ELISA Microplate Reader (Biotek) at 570 nm and was calculated by (test/control) × 100.

### 2.8. miRNA detection in living cells

#### 2.8.1. Fluorescence microscopy

To monitor the expression level of miRNA-21 in DU145, PC3, and LNCaP cells and also analyze cell targeting capability of FAM-Probe21-NMOF-FA complex in PC3 (with high expression folate acid receptor) and A549 (with low expression folate acid receptor) *in vitro*, the cells were cultured in the density of 6 × 10^4^ (in 24-well plates) for 24 h at 37 °C with 5% CO_2_. Then, FAM-Probe21-NMOF-FA or FAM-ScProbe-NMOF-FA complex was applied to the cells for 4 h at 37 °C at 5% CO_2_. Before imaging, the old RPMI medium was removed, and the cells were washed slightly by PBS buffer (pH 7.4). The images were obtained using a microscope from a confocal fluorescence microscope. The fluorescence emission from cells was received in the range of green fluorescence wavelength range (505–540 nm).

#### 2.8.2. Flow cytometry

To quantitative of endogenous miRNA-21 in living cancer cells, flow cytometry was carried out. Briefly, DU145, PC3, and LNCaP cells, with different expression levels of miRNA-21, were seeded in a density of 6 × 10^4^ and then incubated with FAM-Probe21-NMOF-FA or FAM-ScProbe-NMOF-FA complex for 4 h at 37 °C. The cells were collected by using trypsin and washed with PBS two times, and then the fluorescence intensity of each sample was measured by a FACSCalibur flow-cytometer (BD Bioscience; USA), and the fluorescence intensity of each cell was analyzed by Flow Jo.

#### 2.8.3. Reverse transcription quantitative real-time PCR (RT-qPCR)

The total RNA of cells was extracted using Trizol Reagent (Invitrogen, USA) according to the manufacturer's protocol instructions and treated with DNase I, respectively. Total RNA (1 μg) was used to synthesize cDNA using a poly-A polymerase and RevertAid Reverse Transcriptase (Fermentas, Lithuania), and then performed quantitative real-time PCR using a SYBR® Premix Ex Taq II (Takara Bio Inc.) by using ABI 7500 fast real-time PCR system (Applied Biosystems, Foster City, CA, USA), according to the manufacturer's instructions. RNU6 was used as the internal control and all reactions were done in triplicate. The primers and Probe21 used in the following experiment are presented in Table 1.

**Table 1:** List of the real time PCR primers and FAM-Probe21

Gene name	Primer sequence
miRNA-21	5′-TAGCTTATCAGACTGATGTTGA-3′
RNU6	5′-CGCAAGGATGACACGCAAAT-3′
Universal	5′-AACTCAAGGTTCTTCCAGTCACG-3′
FAM-Probe21	5′-/56-FAM/TCAACATCAGTCTGATAAGCTA-3′

## 3. Results

### 3.1. Synthesis and characterization of NMOF (UiO-66-NH_2_), NMOF-FA, and FAM-Probe21-NMOF-FA complex

**Fig. 2:**
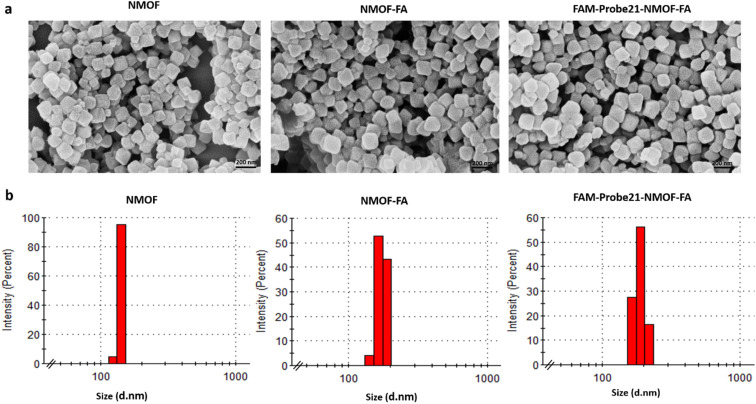
Characterization of NMOFs (UiO-66-NH_2_), NMOF-FA and FAM-Probe21-NMOF-FA using (a) SEM and (b) DLS.

The size and shape of NMOF (UiO-66-NH_2_), NMOF-FA, and FAM-Probe21-NMOF-FA complex were studied using a field emission scanning electron microscope (FE-SEM). As shown in FE-SEM images, the octahedral nanostructure had smooth surfaces in all three nanoparticles (Fig. 2a). Results showed that the particle diameter was 90–170 nm. Images implying that the size and morphology were slightly changed and large after binding FA and FAM-Probe21 on the surface of NMOF. The characterization analyses with dynamic light scattering (DLS) tests of three nanoparticles displayed an average hydrodynamic diameter from 120 to 190 nm with a good small size distribution (Fig. 2b). Consistently, DLS analysis indicated that modification of UIO-66-NH_2_ with FA and Probe21 changed the hydrodynamic size of UIO-66-NH_2_ nanoparticles. Zeta potential data of UIO-66-NH_2_ was equal to −21.8 mV, and altered to −13.2 mV and −13.6 mV after binding FA and Probe21, respectively in PBS. Furthermore, N_2_ adsorption–desorption isotherms are obtained to check the porosity of UIO-66-NH_2_ (Fig. S1†). The Brunauer–Emmett–Teller (BET) results represented that surface area of UIO-66-NH_2_ were significantly reduced following FA and FAM-Probe-21 attachment to NMOF surface (Table 2). These results verified the successful loading of the FA and FAM-probe-21 into the nanocarrier. Moreover, the NMOFs chemical composition was analyzed by energy-dispersive X-ray (EDX) spectroscopy (Fig. S2†) and the data showed that UIO-66-NH_2_ was synthesized successfully. To confirm the crystallinity of NMOF (UiO-66-NH_2_), X-ray diffraction (XRD) spectrum was used. In Fig. S3,† XRD pattern showed a good crystallinity, and purity of NMOF (UiO-66-NH_2_), NMOF-FA and FAM-Probe21-NMOF-FA. The data showed that after modification of UIO-66-NH_2_ with FA and FAM-Probe21, its structure was preserved. In addition, we used FAM-Probe21-NMOF for detection of 1 μM miRNA in cell free condition for four times (Table S1†). Results indicated excellent stability and reproducibility with relative standard deviations (RSDs) of 0.88% and 0.92%, respectively.

**Table 2:** BET and nitrogen adsorption–desorption isotherm data of NMOF (UIO-66-NH_3_), NMOF-FA and FAM-Probe21-NMOF-FA

Samples	BET	Nitrogen adsorption–desorption isotherm			
	Surface area (m^2^ g^−1^)	Pore volume adsorption (cm^3^ g^−1^)	Pore volume desorption (cm^3^ g^−1^)	Pore size adsorption (Å)	Pore size desorption (Å)
UIO-66-NH_2_	706.45	0.22	0.20	21.94	21.57
UIO-66-NH_2_-FA	470.36	0.74	0.77	52.30	53.58
FAM-Probe21-UIO-66-NH_2_-FA	235.88	0.62	0.62	51.54	51.20

### 3.2. *In vitro* fluorescence quenching capability of NMOF (UiO-66-NH_2_) and recovery of fluorescence emission

**Fig. 3:**
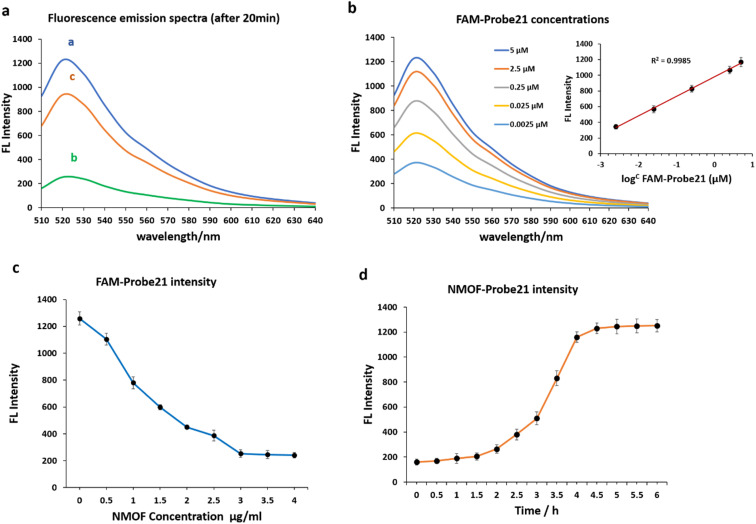
(a) Fluorescence emission spectra of FAM-Probe21 at different conditions: (curve a) FAM-Probe21 in the absence of NMOF (UiO-66-NH_2_), (curve b) FAM-Probe21 in the presence of NMOF (UiO-66-NH_2_). (Curve c) FAM-Probe21 + NMOF (UiO-66-NH_2_) + target miRNA (miR-21). (b) Fluorescence emission spectra of FAM-Probe21 after incubation with varying concentrations of targeted miRNA at room temperature. (c) Effect of dosage of NMOF (UiO-66-NH_2_) on fluorescence intensity of FAM-Probe21. (d) Influence of incubation time between the FAM-Probe21-NMOF complex and the target miRNA on fluorescence intensity.

We examined the fluorescence quenching ability of NMOF (UiO-66-NH_2_) on carboxyfluorescein (FAM) and the release of dye-labeled oligonucleotide (Probe21) from the NMOF (UiO-66-NH_2_) surface following add the complementary miRNA in a homogeneous solution (Fig. 3a).

As shown in curve a, miR-21 probes displayed a strong fluorescence intensity cause of the labeled fluorescein-based fluorophore (FAM). However, upon the addition of NMOF (UiO-66-NH_2_), the fluorescence emission decreased dashingly by 79.29% (curve b), leading to static electronic communication between FAM and NMOF (UiO-66-NH_2_) and indicating the efficient fluorescence quenching capability of NPs. In the presence of target miR-21, the probe bound with its target and detached from the NMOF, which led to fluorescence recovery to 72.87% (curve c).

The concentrations of FAM-Probe21 upon introducing the target miRNAs (1 μM) were also optimized for the best fluorescence recovery efficiency. Fig. 3b shows the optimal recovered fluorescence emission was in 5 μM of FAM-Probe21. To reach high fluorescence quenching efficiency of NPs, the relative amount of Probe21 and NMOF was optimized by assessing the emission spectra of the probe in response to the NPs with different concentrations. Results indicated that in the presence of NMOF with different concentrations (0, 0.5, 1, 1.5, 2, 2.5, 3, 3.5, 4 μg mL^−1^), the optimal fluorescence quenching of FAM-probe-21 was measured at 520 nm and within 20 min (Fig. 3c). The data showed that the addition of 3 μg per mL ≤ of NMOF-FA resulted in complete fluorescence quenching of 5 μM of FAM-Probe21.

Furthermore, our experiments indicated an optimal time for high fluorescence recovery of FAM-Probe21-NMOF complex (3 μg per mL NMOF and 5 μM FAM-Probe21) by introducing the target miRNA (1 μM). As shown in Fig. 3d, the fluorescence intensity was gradually enhanced with the increase of the incubation time till 4 h, after this time, the system reached an equilibrium between FAM-Probe21-NMOF and the FAM-Probe21-NMOF/target complex, resulting in the fluorescence intensity displayed no further enhancement.

### 3.3. *In vitro* sensitivity and specificity of FAM-Probe21-NMOF

**Fig. 4:**
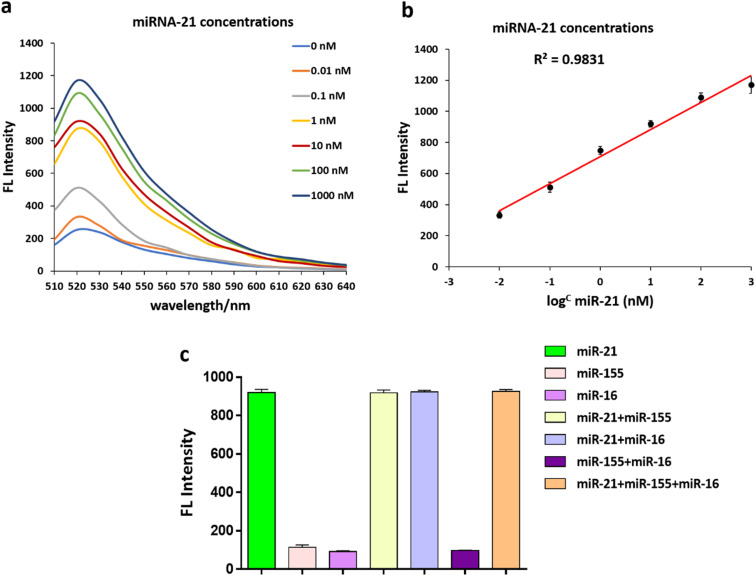
(a) and (b) Fluorescence spectra of the FAM-Probe21-NMOF complex in response to (7) the different concentrations of target miRNAs; the lowest spectra represent the basal fluorescence emission in the complete absence of targets. The concentration of each FAM-Probe21 was 5 μM. (c) The sequence cross-reactivity and specificity of the suggested miRNA sensor.

Under optimal conditions, an additional experiment verified that the fluorescent intensity of the FAM-Probe21-NMOF solution increased with increasing concentration of the target miRNA (miR-21) in the range from 0 to 1000 nM. These results implied the fluorescence intensity of FAM-Probe21-NMOF was logarithmically dependent on the miRNA concentration, with a detection limit of 10 PM (Fig. 4a and b).

To investigate the cross-reactivity and specificity of the miRNA sensor method, three miRNAs (miR-21, miR-155, miR-16) were used and interfering experiments were performed. 1 μM of each miRNA was transferred to a 96-well plate with different combinations of them, and a mixed solution of FAM-Probe21-NMOF was added to each well. As expected, the recovered fluorescence emission the wells containing non-complementary miRNAs (miR-155, miR-16) was very weak in compared with wells containing complementary miRNA (miR-21) (Fig. 4c). Therefore, the FAM-Probe21-NMOF can be potentially used in complex biosensor with high sequence specificity for miR-21.

### 3.4. Biocompatibility of FAM-Probe21-NMOF-FA

**Fig. 5:**
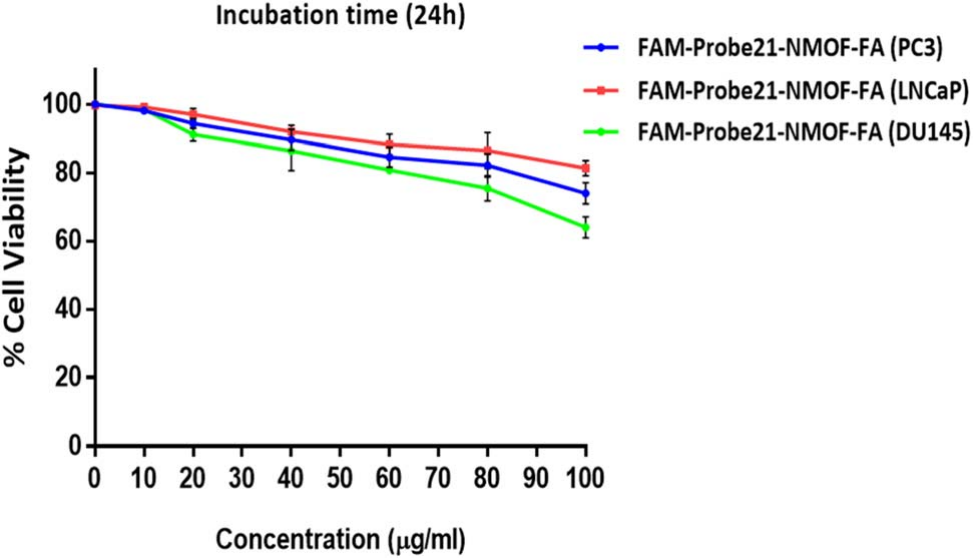
Cytotoxicity test of the FAM-Probe21-NMOF-FA in three cell lines (DU145, PC3, and LNCaP). The viability of cells incubated with different concentrations of FAM-Probe21-NMOF-FA for 24 h were measured by MTT assay. Throughout the present study, the concentration of FAM-Probe21-NMOF-FA was maintained at 10 μg mL^−1^, which ensured ∼100% cell viability. Data are shown as mean ± SD (*n* = 4, *p value < 0.05 between two groups).

The potential cytotoxicity of FAM-Probe21-NMOF-FA in different concentrations (0, 10, 20, 40, 60, 80, 100 μg mL^−1^) was evaluated on DU145, PC3, and LNCaP prostate cancer cells lines by standard MTT assay. The results indicated that in three cancer cell lines, the cell viability could maintain above ∼80% after the treatment of FAM-Probe21-NMOF-FA even at higher concentrations (up to 80 μg mL^−1^) (Fig. 5). In this study, the FAM-Probe21-NMOF-FA concentration was used at 10 μg mL^−1^, below which the cytotoxicity was insignificant. Therefore, the low cytotoxicity property of FAM-Probe21-NMOF-FA suggests this complex as a carrier in probe applications for intracellular biomolecules monitoring.

### 3.5. Cell-specific delivery of FAM-Probe21-NMOF-FA

**Fig. 6:**
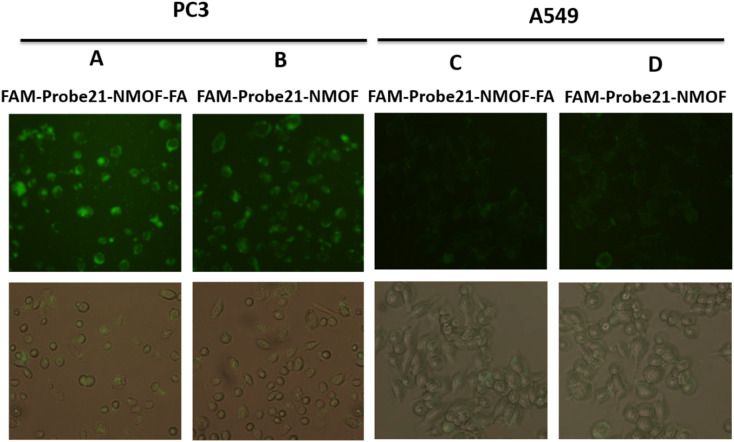
The fluorescence images of PC3 and A549 cells after treating with FAM-Probe21-NMOF-FA or FAM-Probe21-NMOF complex for 4 h. Column A: PC3 + FAM-Probe21-NMOF-FA, column B: PC3 + FAM-Probe21-NMOF, column C: A549 + FAM-Probe21-NMOF-FA, column D: A549 + FAM-Probe21-NMOF. Data are shown as mean ± SD (*n* = 3, *p value < 0.05 between two groups).

In this study, we used FA (folic acid) to reach the cell-specific delivery of FAM-Probe21-NMOF by recognizing the folate receptor overexpressed on the cancer cell membrane. To evaluate the cell-specific delivery of FAM-Probe21-NMOF-FA, A549 cells (with low expression folate acid receptor) and PC3 cells (with high expression folate acid receptor) were treated with FAM-Probe21-NMOF-FA or FAM-Probe21-NMOF complex. As shown in Fig. 6, with the increasing incubation time of FAM-Probe21-NMOF-FA complex treatment, PC3 cells indicated enhancing fluorescence intensity of probe (column A), showing the rising uptake of FAM-Probe21-NMOF-FA. However, the PC3 cells treated with FAM-Probe21-NMOF complex displayed low fluorescence intensity (column B). Also, treated A549 cells with FAM-Probe21-NMOF-FA or FAM-Probe21-NMOF complex showed very low and same fluorescence intensity of probe, due to lack of the folate receptor on the cell (column C and column D).

### 3.6. Detection of intracellular miRNA

**Fig. 7:**
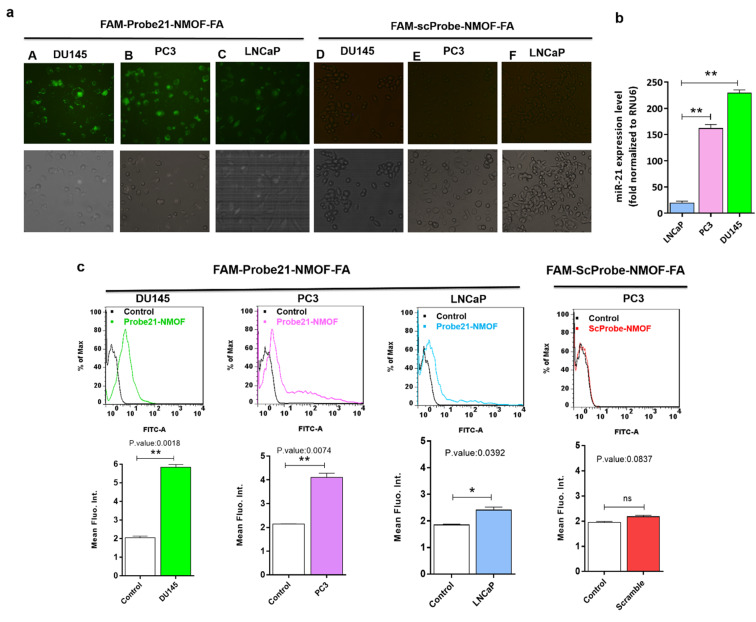
(a) The fluorescence images of DU145, PC3, and LNCaP cells after treating with FAM-Probe21-NMOF-FA or FAM-ScProbe-NMOF-FA complex for 4 h. Column A: DU145 + FAM-Probe21-NMOF-FA, column B: PC3 + FAM-Probe21-NMOF-FA, column C: LNCaP + FAM-Probe21-NMOF-FA, column D: DU145 + FAM-ScProbe-NMOF-FA, column E: PC3 + FAM-ScProbe-NMOF-FA, column F: LNCaP + FAM-ScProbe-NMOF-FA. (b) RT-QPCR shows the expression levels of miRNA-21 in DU145, PC3, and LNCaP. (c) Flow cytometry analysis of DU145, PC3, and LNCaP treated with FAM-PNA21-NMOF, the controls are cells with treatment by FAM-ScProbe-NMOF-FA complex. Data are shown as mean ± SD (*n* = 3, *p value < 0.05 between two groups).

In order to study the capability of the FAM-Probe21-NMOF-FA complex for monitoring intracellular miRNA-21 in DU145, PC3, and LNCaP cells, the cell imaging was performed by confocal microscopy following incubation with 10 μg per mL of FAM-Probe21-NMOF-FA complex or FAM-ScProbe-NMOF-FA (scrambled miR-21 with a mismatched sequence as a control) after 4 h. As shown in Fig. 7a, the fluorescence signal in DU145 was stronger than PC3 and LNCaP, due to the different expression levels of miRNA-21 in DU145 (column A), PC3 (column B), and LNCaP cell lines (column C). And also, in the control experiments, the FAM-ScProbe-NMOF-FA complex induced no obvious fluorescence signal in all three treated cells (column D, column E and column F). Consistently, RT-QPCR confirmed that DU145 expressed a relatively higher level of miRNA-21 than PC3 and LNCaP (Fig. 7b). To further confirm the detection outcomes, Flow cytometry analysis of intracellular miRNA-21 was used in DU145, PC3, and LNCaP cells, following FAM-Probe21-NMOF-FA complex or FAM-ScProbe-NMOF-FA incubation. The results were consistent with previous data and exhibited that the fluorescence intensity in DU145 was stronger than PC3 and LNCaP compared with control cells (Fig. 7c).

**Fig. 8:**
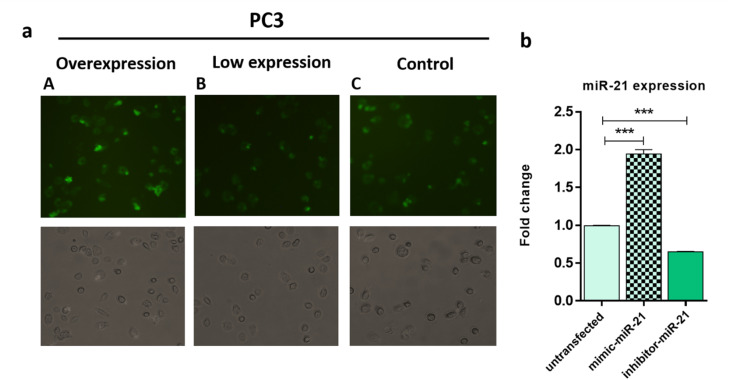
(a) Detection of miR-21 in treated PC3 with difference expression of miR-21 by using FAM-Probe21-NMOF-FA. PC3 cells were incubated with miRNA inhibitor or miRNA-21 mimic for 24 h, then the cells were treated with the FAM-Probe21-NMOF-FA complex for 4 h. (b) RT-QPCR shows difference expression levels of miR-21 in treated cells by miRNA inhibitor or miRNA-21 mimic compared with control cells. Data are shown as mean ± SD (*n* = 3, *p value < 0.05 between two groups).

To confirm the sensitivity function of the FAM-Probe21-NMOF-FA complex, the expression level of miRNA-21 was altered in PC3 cells by using miRNA21-antisense or miRNA21-mimic, followed by incubating with the FAM-Probe21-NMOF-FA complex and detected by confocal microscopy, subsequently (Fig. 8a). Treated PC3 cells by miRNA21-mimic exhibited a strong fluorescence signal of FAM-Probe21-NMOF-FA complex (column A), while there was weak fluorescence intensity in PC3 cells treated with miRNA21-antisense (column B) in compared to un-transfected cells (column C). Furthermore, RT-QPCR analysis was performed to analyze the miRNA-21 expression level in the treated cells (Fig. 8b). Results indicated that the expression level of miRNA-21 down-regulated about 35.2% in the treated PC3 cells with miRNA21-antisense, in comparison the incubated cells with miRNA21-mimic led to 188.8% up-regulation of the miRNA-21 expression level in compared to un-transfected cells. These achievements exhibited that the proposed miRNA sensor could be well used to monitor the dynamic change of miRNA-21 expression in living cancer cells and normal cells.

## 4. Discussion

MiRNAs are known as key players in the development and progression of diseases such as cardiovascular diseases^39^ and cancer.^40^ Pieces of evidence showed that miRNAs enable to prediction of the clinical outcome of patients with prostate cancer,^41^ and monitoring the differential expression of particular of them in cells, can be a helpful tool for making the diagnosis and prognosis of this cancer.^42^ In prostate cancer, the expression level of miR-21, alongside other biomarkers, has been associated with the pathological stage, metastasis, and progression of diseases.^43^ So, providing appropriate methods for miR-21 sensing in prostate cancer cells compared with normal cells, represents a promising strategy for diagnosing and treating of prostate cancer.^44^ Folate receptors are typical receptors on the surface of cells which overexpressed in cancer cells.^45^ Recently, folate conjugated biosensors and nanoparticles have been developed for specific targeting of cancer cells.^46,47^

There is no selective targeting for monitoring expression level of miR-21 in prostate cancer cells by using a delivery system based on UIO-66-NH_2_. In this study, a biosensor was designed in which folate was conjugated to UIO-66-NH_2_ nanoparticles and FAM-Probe-21 assembled to them to detect miR-21 in prostate cancer cell lines. UIO-66-NH_2_ has various unique properties, such as flexible porosity and intrinsic fluorescence quenching which are the reasons for its superior use in biosensors compared to other conventional nanostructures.^34–36^ Previously, NMOF has been applied for successful deliver of fluorouracil (5-FU) in HePG-2 cells for fluorescence imaging and cancer therapy.^47^ Also, Wu and *et al.* studied a fluorophore-labeled probes that are firmly conjugated with an NMOF as a fluorescence quencher to detect miR-21 in the breast cancer cell lines.^48^ Compared to the mentioned study, we improved the characteristics of the biosensor through binding of folate to NH_2_ surface of the NMOF^49^ which enhanced delivery of FAM-Probe21-NMOF-FA to prostate cancer cells by targeting the folate receptor (FR). An *in vitro* study was established using folic acid (FA)–poly(ethylene glycol)-functionalized MoS_2_ nanosheets to delivery miR-21 probe in MCF-7 and HeLa cells.^50^ The authors demonstrated that the FA conjugation could protect the probes and improve cancer cell transfection efficiency. Although folic acid conjugated Nano carriers has been used for targeting the delivery of drugs or genes into cancer cells,^52^ to our best of knowledge, this study is the first to illustrate the monitoring endogenous miRNA expression *via* FA-NMOF based nanoprobe in prostate cancer cells. Guan and *et al.* reported that miR-21 promotes PCa cell proliferation and colony formation and also showed that miR-21 expression level in DU145 cell line, as an invasive prostate cell, is higher than other prostate cells (PC3 and LNCaP).^51^ Our results indicated that the studied biosensor could detect the altered expression of miR-21 in prostate cells with high sensitivity.

NMOF-based miRNA detection in the present work is free from the laborious and expensive isolation or amplification procedures, which is a crucial advantage for the *in situ* analysis of living cells. We speculate that the formulated FA-NMOF-miR-21 system represents a potential approach for *in situ* detection of intracellular miRNA for early diagnostics and treatment of diseases.

## 5. Conclusion

In summary, FAM-Probe21-NMOF-FA based on UIO-66-NH_2_ was synthesized and used as a biosensor application to monitor miR-21 in prostate cancer cells. MTT assay showed that the folic acid conjugated FAM-Probe21-NMOF was non-toxic for cells. *In vitro* cellular and bio imaging study showed an enhancement uptake of FAM-Probe21-NMOF-FA and fluorescent intensity due to more detection of miR-21 in cells, through targeting folate receptors on the cancer cells by folic acid conjugated on FAM-Probe21-NMOF complex. Therefore, these achievements suggest that folic acid conjugated FAM-Probe21-NMOF complex may represent a promising diagnostic strategy for sensing miRNAs in prostate cancer cell lines in different stages and different expression of folate receptor.

## Author contributions

MA made the conception and design. MJR and MK participated in the development of the methodology. MA and FY performed the experiments. MA and FY analyzed the data and wrote the manuscript. MJR and MK reviewed and/or revised the manuscript. All authors read and approved the final manuscript.

## Conflicts of interest

The authors declare no conflicts of interest.

## Supplementary Material

RA-012-D2RA04959G-s001
