# Supplementary material for: Metal–organic frameworks-based biosensor for microRNA detection in prostate cancer cell lines
Source: RSC Adv. 2022 Dec 7;12(54):35170–80. doi: 10.1039/d2ra04959g (PMC9727830; doi:10.1039/d2ra04959g)
Supplement: RA-012-D2RA04959G-s001 [file RA-012-D2RA04959G-s001.pdf]

Electronic Supplementary Material (ESI) for RSC Advances.
This journal is © The Royal Society of Chemistry 2022

Supplementary Date

Metal–organic frameworks-based biosensor for MicroRNA detection in prostate cancer cell lines

Milad Ahmadi Najafabadi [1], Fatemeh Yousefi[2], Mohammad J. Rasaee[1*], Masoud Soleimani[3*], Mahmood Kazemzad[4]

1 Department of Medical Biothecnology, Faculty of Medical Sciences, Tarbiat Modares University, Tehran, Iran

2 Department of Department of Genetics, Faculty of Biological Sciences, Tarbiat Modares University, Tehran, Iran

3 Department of Hematology, Faculty of Medical Sciences, Tarbiat Modares University, Tehran, Iran

4 Material & Energy Center (MERC), Tehran, Iran

*Corresponding author e-mail: rasaee_mj@yahoo.com; Soleimani.masoud@gmail.com

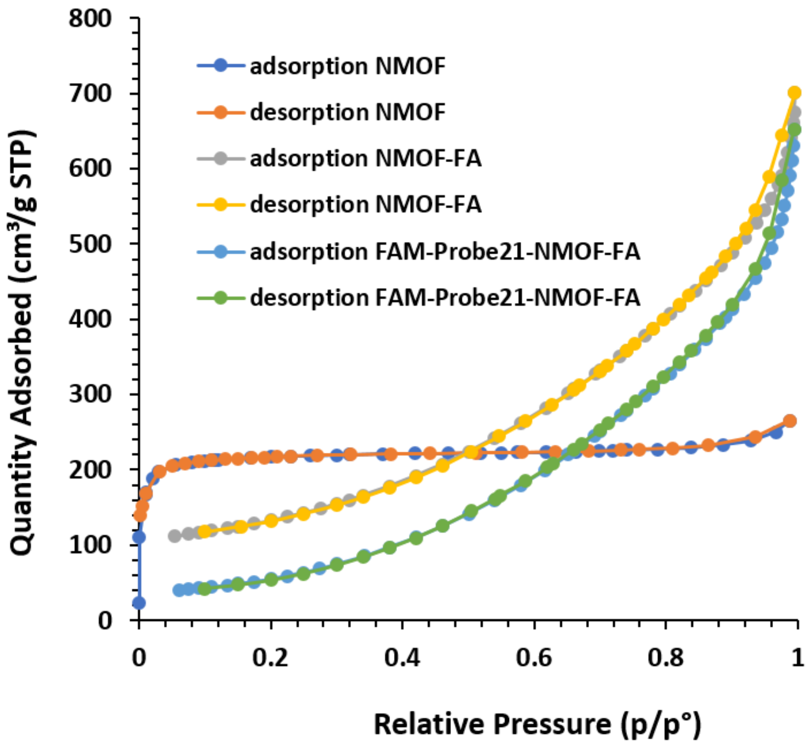

Figure S1: Nitrogen adsorption-desorption isotherms of NMOF(UIO-66-NH2), NMOF-FA and Probe21-NMOF-FA.

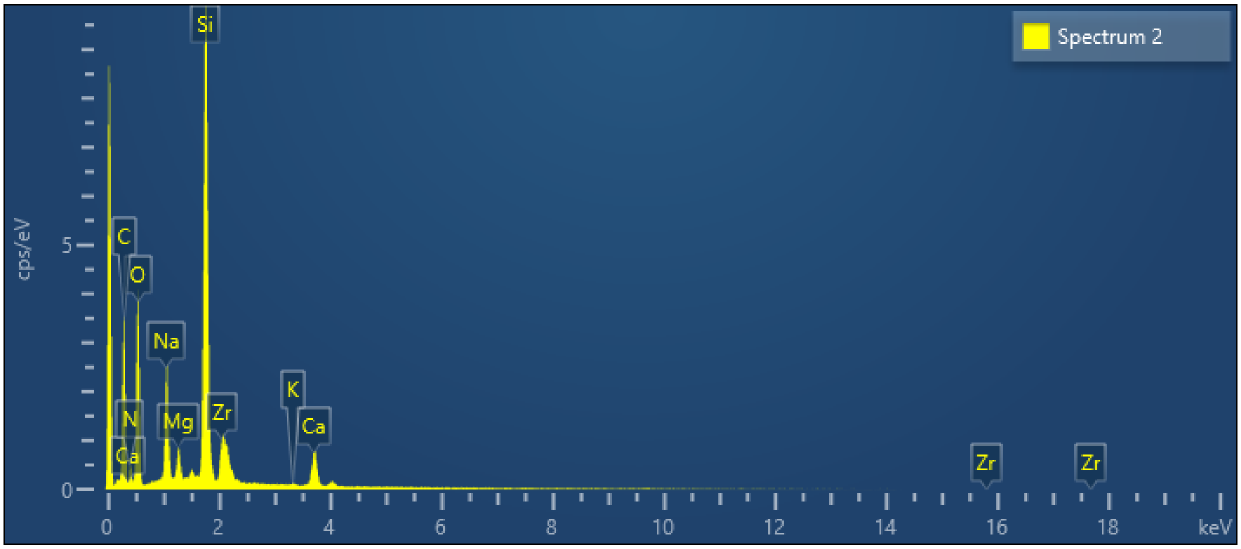

Figure S2: Energy-Dispersive X-ray (EDX) spectroscopy of UIO-66-NH2

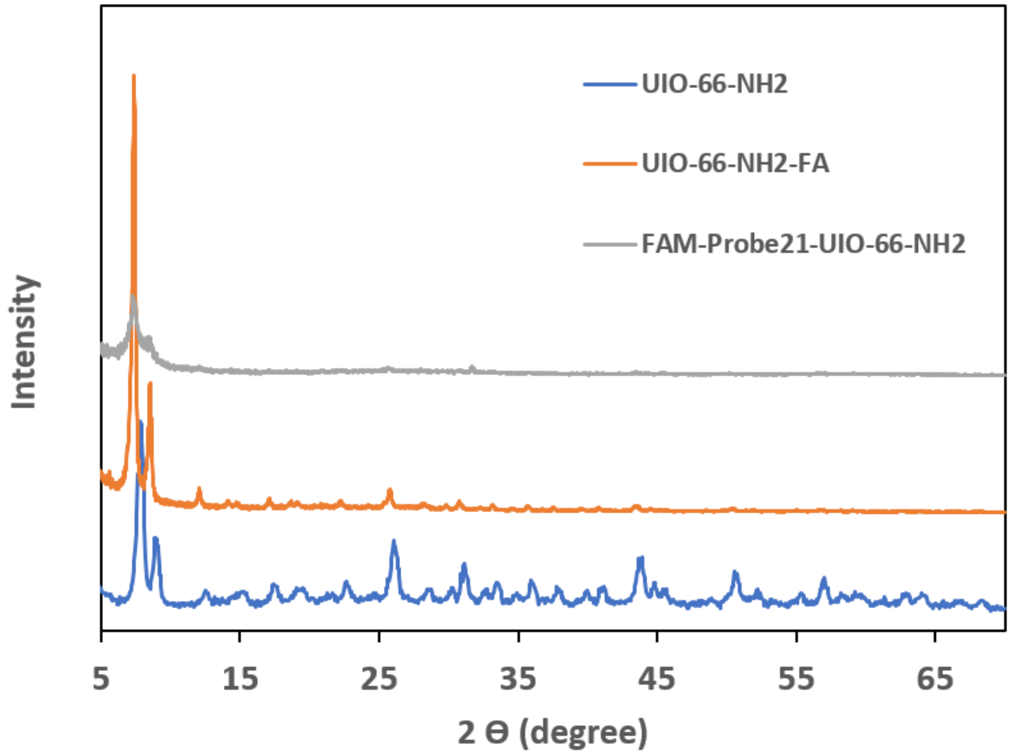

Figure S3: X-ray diffraction patterns (EXRD) of NMOF (UIO-66-NH2), UIO-66-NH2-FA and Probe21-UIO-66-NH2-FA.

Table S1: Experimental data for the stability and reproducibility of FAM-Probe21-NMOF. Concentration of NMOF, FAM-Probe21 and miR-21 were 3 µg/mL, 5 µM and 1 µM, respectively.

**Stability**					
**Test**	**1**	**2**	**3**	**4**	**RSD(%)**
**Fluorescence intensity**	1226.4	1212.4	1205.1	1202.5	0.88%
**Reproducibility**					
**Biosensor**	**1**	**2**	**3**	**4**	**RSD(%)**
**Fluorescence intensity**	1226.6	1207.6	1218.8	1201.5	0.92%
